# Yield-stress transition in suspensions of deformable droplets

**DOI:** 10.1126/sciadv.adf8106

**Published:** 2023-05-31

**Authors:** Giuseppe Negro, Livio Nicola Carenza, Giuseppe Gonnella, Fraser Mackay, Alexander Morozov, Davide Marenduzzo

**Affiliations:** ^1^ Dipartimento di Fisica, Universitá degli Studi di Bari and INFN, Sezione di Bari, via Amendola 173, Bari I-70126, Italy.; ^2^ Instituut-Lorentz, Universiteit Leiden, P.O. Box 9506, 2300 RA Leiden, Netherlands.; ^3^ School of Physics and Astronomy (SUPA), University of Edinburgh, Peter Guthrie Tait Road, Edinburgh EH9 3FD, UK.

## Abstract

Yield-stress materials, which require a sufficiently large forcing to flow, are currently ill-understood theoretically. To gain insight into their yielding transition, we study numerically the rheology of a suspension of deformable droplets in 2D. We show that the suspension displays yield-stress behavior, with droplets remaining motionless below a critical body-force. In this phase, droplets jam to form an amorphous structure, whereas they order in the flowing phase. Yielding is linked to a percolation transition in the contacts of droplet-droplet overlaps and requires strict conservation of the droplet area to exist. Close to the transition, we find strong oscillations in the droplet motion that resemble those found experimentally in confined colloidal glasses. We show that even when droplets are static, the underlying solvent moves by permeation so that the viscosity of the composite system is never truly infinite, and its value ceases to be a bulk material property of the system.

## Teaser

Numerical simulations of 2D phase field models show that deformable suspensions display the hallmark of yield-stress fluids.

## INTRODUCTION

Yield-stress fluids are materials that flow only when subject to a sufficiently large stress or external forcing (*1*, *2*). The critical stress above which there is flow is known as the yield stress. Examples of yield-stress fluids abound in everyday materials and include toothpaste, whipping or shaving cream, mayonnaise, and cement. The defining property of an ideal yield-stress fluid is that the apparent viscosity should be infinite below yielding so that the yield stress should mark a transition between a solid-like and a fluid-like regime. Nevertheless, in practice, it is often arduous to distinguish this behavior from that of a strongly shear-thinning fluid for which the viscosity drops by orders of magnitude at the yielding point, such that the material always flows albeit very slowly under any external forcing, however small (*2*).

Phenomenological theories for yield-stress fluids typically assume a non-Newtonian and nonlinear relation between the shear stress σ and the shear rate (or velocity gradient) $\dot{\gamma }$. A popular model is the Herschel-Bulkley one (*3*), which is based on the generic equation $\sigma =\sigma_{y}+\eta_{\infty }{\dot{\gamma }}^{n}$, with σ*_y_* being the yield stress, η_∞_ being a material parameter, and *n* being a generic exponent found by fitting experimental data and smaller than 1 for shear-thinning fluids. Phenomenological models like this are extremely useful to analyze and compare experiments, but, by their nature, they do not address the fundamental physical mechanisms underlying the existence of a yield stress.

Yield-stress fluids can be characterized according the softness of their constituents (*2*) and range from foams (*4*–*14*) to suspensions of nearly-hard colloidal spheres (e.g., spherical particles stabilized sterically with a thin polymer layer) (*15*–*19*). In all cases, at large-enough particle concentrations, such that the system is the jammed, or glassy, phase, these materials are experimentally known to undergo a yielding transition. They also display soft glassy rheology, as described by the Herschel-Bulkley model (*20*–*24*). In colloidal fluids, rheological experiments further show that the effective viscosity of the system becomes very large and possibly diverges (*2*, *16*). A confounding factor hampering a conclusive demonstration of ideal yield-stress behavior in experiments is that the solid-like phase in a colloidal glass is amorphous, and the fundamental physics of the amorphous state is not fully understood (*2*). In addition, as we show to be relevant here, colloidal glasses or foams are composite materials so that the behavior of the dispersed particles and the underlying solvent may differ, thereby complicating the picture.

Here, we consider a generic universal model system for a yield-stress fluid: a two dimensional suspension of soft deformable droplets embedded in a Newtonian fluid (*25*–*27*). We focus on the case of a deformable suspension with intermediate droplet density so that a physical realization of our system is provided, for instance, by a stabilized oil-in-water emulsion. Note that in other parameter regimes, not explicitly considered here, the same model can effectively describe colloidal suspensions, when particle deformation is negligible, or foams, for sufficiently large droplet density.

By performing extensive numerical simulations, we show that deformable suspensions display the hallmark of yield-stress fluids, as the droplets are immobile even when subjected to a (small) pressure difference or forcing. In this immobile phase, the droplets are arranged in an amorphous pattern, and the network of droplet-droplet contacts, or overlaps, percolates. These overlaps may be interpreted as the soft analog of frictional contacts, which are known to play a crucial role in colloidal rheology (*28*). Upon yielding, contact percolation is lost, while droplets order as they flow. We find that even in the phase where droplets are static, the solvent flows by permeation, meaning that the viscosity of the overall system is never truly infinite. Close to the yielding point, sustained velocity oscillations occur, similarly to what was found experimentally in flowing colloidal fluids close to the glass transition (*29*). Our results allow us to gain more insight into the microscopic physical mechanisms underlying the yielding transition. In our system, the latter is controlled by an inverse Bingham number, measuring the ratio between viscous and interfacial forces. Notably, this is the same number that controls discontinuous shear thinning at larger forces (*25*, *26*, *30*), while differing from the capillary number controlling foam rheology (*7*). Last, our simulations suggest that yielding disappears when the droplet area is not strictly conserved, suggesting that systems featuring evaporation-condensation phenomena such as evaporating emulsions or systems where droplets do not have a fixed size, such as microgels (*31*, *32*) or cell monolayers (*33*) can evade yield-stress behavior and flow under any forcing.

## RESULTS

### A multiphase field model to study the rheology of deformable droplets

**Fig. 1.**
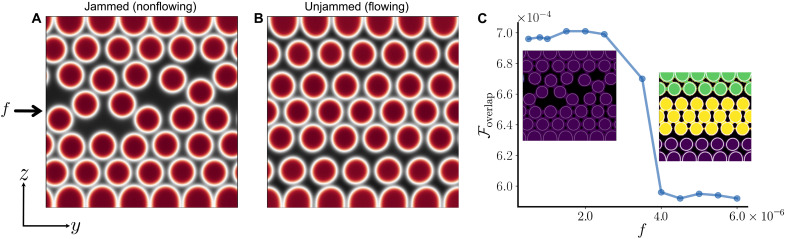
Yielding transition in the conserved model. (**A** and **B**) Color map of ϕ = ∑*_i_* ‍ ϕ*_i_* for *f* < *f_c_* [*f* = 2.0 × 10^−6^ in (A)] and *f* > *f_c_* [*f* = 4.0 × 10^−6^ in (B)], for the conserved model. Black and red regions correspond to ϕ = 0 and ϕ = 2, respectively. (**C**) Free energy of overlaps (see text) as a function of body-force *f*. The insets of (C) show clusters of contacting droplets, resulting from a density-based spatial clustering analysis on the free energy of overlaps (see the Supplementary Materials). Different colors correspond to different clusters. Left and right inset correspond to the configuration shown in (A) and (B), respectively. Movies of the dynamics corresponding to (A) and (B) can be seen in movies S1 and S2, respectively (see the Supplementary Materials).

To study the rheology of our soft droplet suspension, we work in two dimensions (Fig. 1) and consider two models: The first strictly conserves the area of each droplet, and the second allows it to fluctuate around a target value, for instance, because of evaporation or condensation phenomena. We refer to these as the conserved and nonconserved model, respectively. In both cases, the *N* droplets in the system are noncoalescing, and we ensure this by describing them in terms of *N* distinct phase fields, ϕ*_i_*, with *i* = 1, …, *N*. The hydrodynamics of the suspension can then be studied by following the coupled evolution of the phase field variables and of the velocity field **v** of the underlying solvent. The use of phase field means that lubrication forces, which are notoriously challenging to accurately account for in simulations (*28*, *34*) are altogether absent. At the same time, overlaps between different phase fields mimic frictional forces, which are known to play a crucial role in colloidal rheology (*28*).

The thermodynamics of the conserved model is governed by a free energy F whose density is

$$\sum_{i}^{N}\frac{\alpha }{4}\phi_{i}^{2}{\left(\phi_{i}-\phi_{0}\right)}^{2}+\frac{K}{2}\sum_{i}^{N}{\left(\nabla \phi_{i}\right)}^{2}+\sum_{i,j,i<j}\epsilon \phi_{i}^{2}\phi_{j}^{2}$$

Here, the first two terms favor the formation of circular droplets with ϕ*_i_* ≃ ϕ_0_ in their interior and ϕ*_i_* ≃ 0 outside. The material constants α and *K* determine the surface tension $\gamma =\sqrt{8K\alpha /9}$ and the interfacial thickness $\xi =\sqrt{2K/\alpha }$ of the droplets (*35*). The term proportional to ε > 0 describes soft repulsion pushing droplets apart when overlapping. The phase field variables evolve according to a set of coupled Cahn-Hilliard equations

$\frac{\partial \phi_{i}}{\partial t}+v\;\cdot \nabla \phi_{i}=M\nabla^{2}\mu_{i}$ where *M* is the mobility and μ*_i_* = δF/δϕ*_i_* is the chemical potential of the *i*-th droplet. The flow obeys the Navier-Stokes equation

$\rho \left(\frac{\partial }{\partial t}+v\;\cdot \nabla \right)v=-\nabla p+f^{th}+\eta_{0}\nabla^{2}v+fe_{y}$ where ρ indicates the total fluid density, *p* denotes the hydrodynamic pressure, and η_0_ is the solvent viscosity (see the Supplementary Materials). The term **f***^th^* = −∑*_i_*‍ϕ*_i_*∇μ*_i_* stands for the internal thermodynamic force field due to the presence of nontrivial compositional order parameters, while 𝑓 is the magnitude of the body-force, which we take along the horizontal direction (Fig. 1A).

In our second model for the nonconserved concentration field, the free energy *F* is supplemented by an additional term

$F_{\mathrm{constraint}}=\lambda {\left(1-\frac{1}{\pi R^{2}\phi_{0}^{2}}\int \;dydz\;\phi_{i}^{2}\right)}^{2}$ with λ > 0 being a constant that quantifies droplet compressibility and provides a soft constraint for the droplet area. The phase fields evolve according to a relaxational and overdamped dynamics defined by

$\frac{\partial \phi_{i}}{\partial t}+v\;\cdot \nabla \phi_{i}=-\frac{1}{\text{Γ}}\frac{\delta F^{\prime }}{\delta \phi_{i}}$ where Γ is a friction-like parameter and F^′^ = F + F_constraint_. The equation for **v** is still given by Eq. 3.

The dynamics are integrated with a parallel hybrid lattice Boltzmann (LB) approach (*36*–*40*) where Eq. 3 is solved by a LB algorithm and Eqs. 2 and 5 are solved by finite difference methods. We consider flow in a channel with no-slip boundary conditions at the top and bottom walls, driven by a fixed pressure difference along the *y* direction, leading to Poiseuille, parabolic flow for a Newtonian fluid. At the walls, neutral wetting boundary conditions are imposed on each droplet, with no flux at the boundaries and droplets forming an angle of π/2 with the wall surface (Eq. 6). Droplets are initially randomly positioned, and large overlaps are removed by allowing the system to equilibrate before applying the pressure-driven flow via the external body force. For more details and a full list of parameters used, see the Supplementary Materials.

### Monodisperse droplet suspensions display yield-stress behavior and permeation

**Fig. 2.**
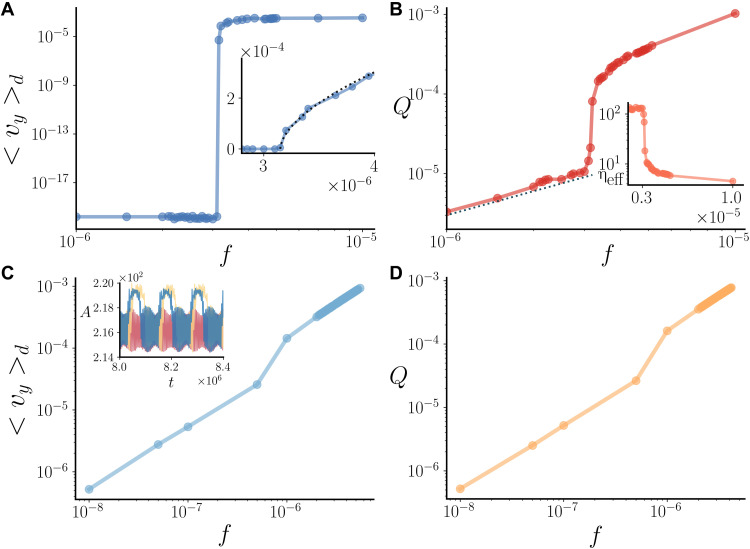
Flow behavior in the conserved and nonconserved models. (**A** and **B**) Average droplet velocity (A) and throughput flow (B) for the conserved model. The inset of (A) shows the mean droplet speed close to criticality and the result of the fit (dashed line) with the function ⟨*v*⟩ ∝ (*f* − *f_c_*)^β^, with β ≃ 0.54. The inset of (B) shows the effective viscosity η_eff_ as a function of the body-force *f*. (**C** and **D**) Average droplet velocity (C) and throughput flow (D) for the nonconserved model. The inset of (C) shows the area of three nearby droplets versus time for *f* = 1.0 × 10^−6^.

We first study the rheological response of a droplet suspension (with packing fraction ϕ ≃ 0.5) in the conserved model. A key result is that there exists a critical body-force *f_c_* separating two fundamentally different behaviors. For small forcing (Figs. 1A and 2A), the suspended droplets are jammed and settle into a stationary nonflowing configuration where they are immobile for the whole duration of the numerical experiment [∼ 𝒪(10^8^) iterations]. The snapshot shown in Fig. 1A shows a typical droplet configuration for this regime. For larger 𝑓, there is a subtle morphological rearrangement of the droplets (Fig. 1B), which is accompanied by a yielding transition, as droplets now steadily move (Fig. 2A). The snapshot shown in Fig. 1B shows a typical late time configuration, which is traveling from left to right at a fixed velocity (Fig. 2A). An inspection of the configurations shows that while the nonflowing state is amorphous (Fig. 1A), in the flowing state, droplets order (Fig. 1B); see the Supplementary Materials for a quantification of flow-induced ordering. This morphological adjustment is accompanied by a fundamental change in the patterns of contacts, or overlaps, between droplets. As shown in the left inset of Fig. 1C, such overlaps create a percolating network in the nonflowing state, whereas after yielding, contacts no longer percolate along the flow gradient direction (right inset of Fig. 1C and fig. S1). Because, in our model, the energetic cost of two droplets overlapping, e.g., *i* and *j*, is proportional to $\epsilon \phi_{i}^{2}\phi_{j}^{2}$, the change in droplet contacts can be quantified by plotting the overlap free energy ($F_{\mathrm{overlap}}=\epsilon \int \;dydz\sum_{i,j}\phi_{i}^{2}\phi_{j}^{2}$) as a function of body-force (Fig. 1C): This quantity drops sharply at the yielding transition, corresponding to the loss of contacts between droplets near the wall (right inset). As discussed in more detail below, another key feature is that droplets need to deform at least transiently when the system yields (see the Supplementary Materials).

To quantify the yielding transition, we compute two quantities: (i) the mean velocity of the droplets’ center of mass ⟨*v_y_*⟩*_d_*, (Fig. 2, A and C) and (ii) the throughput flow *Q* = ∫ ‍ *dydz v_y_* (Fig. 2, B and D). While ⟨*v_y_*⟩*_d_* quantifies the motility of the suspended particles, *Q* can be used to compute the effective viscosity of the suspension, η_e*ff*_. The latter quantity can be estimated as $\eta_{eff}=\eta_{0}\frac{Q_{0}}{Q}$, where $Q_{0}=\frac{fL^{3}}{12\eta_{0}}$ is the throughput flow of a Newtonian fluid with viscosity η_0_ subject to a body-force *f* and *L* is the channel width. The yield-stress behavior is apparent from the plot of ⟨*v_y_*⟩*_d_* in Fig. 2B. Close to criticality, the mean droplet speed behaves as ∼(*f* − *f_c_*)^β^, with β ≃ 0.54. The phenomenology resembles that of the Prandtl-Thomlinson model (where β = 1/2), which describes a particle in a dashboard potential and provides a simple microscopic model for dry friction (*41*).

Even in the nonflowing phase in which droplets are at rest, the underlying solvent flows (Fig. 2B and fig. S1): *Q* is nonzero for all values of *f*. In more detail, we find that there is a well-defined linear regime at small forcing, which corresponds to a high but finite effective viscosity (Fig. 2B, inset). In stark contrast, yield-stress fluid under shear exhibit wall slip and an infinite effective viscosity (*2*). This shows that the exact value of η_e*ff*_ depends on the geometry of the system and hence can no longer be viewed as one of its bulk material property. The flow at 0 < *f* < *f_c_* is purely permeative, as the solvent flows through an immobile network of jammed droplets. The distinct behavior of the droplet and solvent components in the suspension is instructive and shows that the composite material behaves in a more complex way than what would be predicted for an ideal single-phase yield-stress fluid. In our conserved model, yielding can therefore be viewed as a continuous transition between a permeation regime with jammed amorphous droplets where solvent flows mainly around them and a flowing ordered phase. In the latter phase, the flow is plug-like (*25*), as found experimentally for colloidal suspensions in a pressure-driven flow (*42*).

### The yielding transition disappears in the nonconserved model

It is interesting to contrast the behavior we have just discussed with that of the nonconserved model, where evaporation and condensation effects are included. Unexpectedly, replacing strict area conservation with a soft constraint leads to a complete loss of yield-stress behavior (Fig. 2, C and D). In the nonconserved model, droplets flow at any value of the forcing, however small, so that it is not possible to define a yield stress. While a yielding-like behavior can still be observed as a smooth crossover, there is no longer a singularity in the droplet velocity curve (Fig. 2C). An analysis of the area of each droplet show that the droplet motion is accompanied by area oscillations whose magnitude is controlled by λ, signaling that motion occurs via evaporation-condensation (Fig. 2C, inset). The behavior of the throughput flow mirrors that of the droplet velocity in this nonconserved model (Fig. 2D).

More insight into the fundamental difference between the conserved and nonconserved models can be gained by analyzing the behavior of a single droplet at a solid wall under an external forcing and with neutral wetting boundary conditions (fig. S5 and movie S3). While, in the conserved model, the droplet sticks to the wall and requires a finite forcing to start moving, in the nonconserved model, evaporation and condensation provide another pathway for contact line motion (*43*), and the droplet drifts along the wall for any value of the forcing. Therefore, besides the presence of a percolating network of droplet overlaps (Fig. 1), the existence of a well-defined yielding transition also requires a suitable behavior of droplets close to the wall.

### Close to yielding, the suspension undergoes oscillatory stick-slip motion

**Fig. 3.**
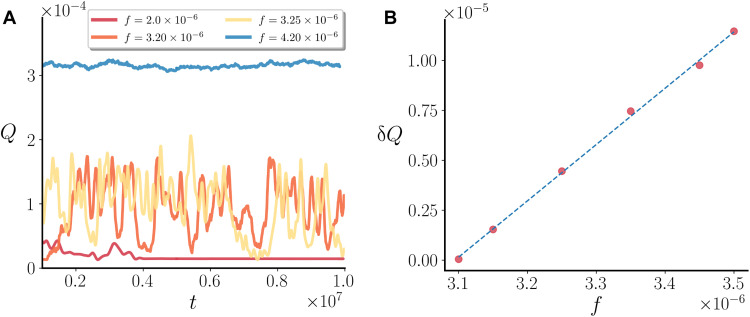
Oscillations near the yielding transition. (**A**) Throughput flow versus time, for the conserved model, for different values of *f* near *f_c_* = 3.15 × 10^−6^. (**B**) Plot of the variance of the oscillations as a function of *f*.

To understand the microscopic mechanism underlying yielding in the conserved model more deeply, we now consider their dynamics close to *f_c_*. Just after yielding, we find a “stick-slip” behavior where the emulsion alternates between plug-like motion, where droplets flow, and stationary spells, where they are almost jammed (movie S4). The throughput solvent flow and the average droplet velocity both show irregular oscillations over time (red and orange curves in Fig. 3A). The average variance (or amplitude) of the stochastic oscillations increases with the forcing and approaches zero at *f_c_* (Fig. 3B).

This behavior is reminiscent of that found in velocity oscillations of colloidal glasses close to the yielding transition (*29*). There are some key qualitative analogies between the two cases. In both systems, the nonflowing and flowing states subtly differ in the typical particle configuration. In our nonflowing emulsions, overlaps between droplets abound and create a nearly percolating chain through the system, just like frictional contacts for hard-sphere colloids. Instead, in the flowing states, there are gaps between most particles (*29*). Analyzing the dynamics in more detail reveals an important distinction. In our system, instantaneous yielding events, i.e., transitions from jammed to flowing states, are typically accompanied by a sudden change in behavior in the deformation free energy $F_{\mathrm{def}}=\frac{K}{2}\int \sum_{i}{\left(\nabla \phi_{i}\right)}^{2}dydz$ (see the Supplementary Materials). This suggests that yielding in our deformable suspensions requires a transient change in droplet shape, which is instead essentially fixed for colloids.

### Polydisperse suspensions yield at larger forcing

So far, we have only considered monodisperse suspensions, where all droplets have the same size. In this section, we explore the effect of polydispersity by considering a bidisperse mixture, where the droplet size of one component is twice as large as that of the other. In this context, the smaller component can be seen as impurities in an otherwise homogeneous system: In a colloidal system, the corresponding case leads to substantial quantitative changes in the material behavior (*44*). In the context of foams, bidispersity was considered theoretically in (*10*): In that regime, its effect on the yield stress was predicted to be negligible.

**Fig. 4.**
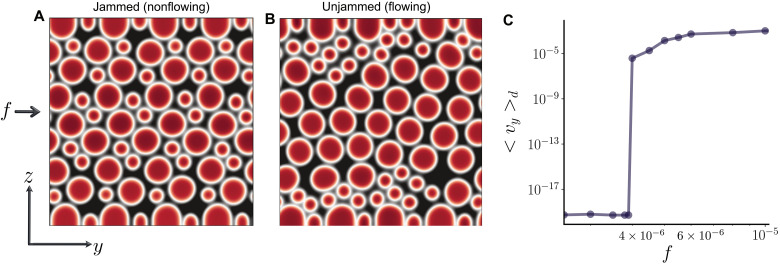
Yielding of bidisperse suspensions. (**A** and **B**) Color map of ϕ = ∑*_i_* ‍ ϕ*_i_* for *f* < *f_c_* [*f* = 2.0 × 10^−6^ in (A)] and *f* > *f_c_* [*f* = 6.0 × 10^−6^ in (B)]. (**C**) Average droplet velocity as a function of the body-force *f*.

Figure 4 (A and B) shows two typical configurations, for low and high body-force, respectively. Analogously to what is observed in the monodisperse case, for low values of the body-force, the droplets are jammed and immobile (Fig. 4, A and C). Here, the smaller droplets tend to sit in the interstitial space between the network of larger droplets. As the body-force is increased, a transition to a flowing state is observed, as signaled by the jump of the mean droplets velocity ⟨*v_y_*⟩*_d_* in Fig. 4C. The transition is once more accompanied by a morphological rearrangement, where the droplets of the two species migrate to different regions of the system: The large droplets move toward the center of the channel, while the small ones relocate close to the walls, effectively creating a lubricating layer (Fig. 4B). Quantitatively, the yield stress transition moves to higher values of the body-force compared to the monodisperse case ($f_{c}^{bd}=4.0\times 10^{-6}$ versus *f_c_* = 3.15 × 10^−6^ for the same packing fraction ϕ = 0.5). This is due to the fact that the smaller droplets fit snugly between larger particles, effectively thickening the percolating network of contacts. Therefore, for the suspension to flow, the forcing has to be large enough to disrupt this thickened network. The dynamics of the system above the yielding transition is shown in fig. S6, which shows how the percolating contact networks is lost and gives way to size-dependent segregation along the channel, as also observed in some cases in non-Brownian colloidal suspensions (*45*).

### Scaling analysis of the yielding transition

**Fig. 5.**
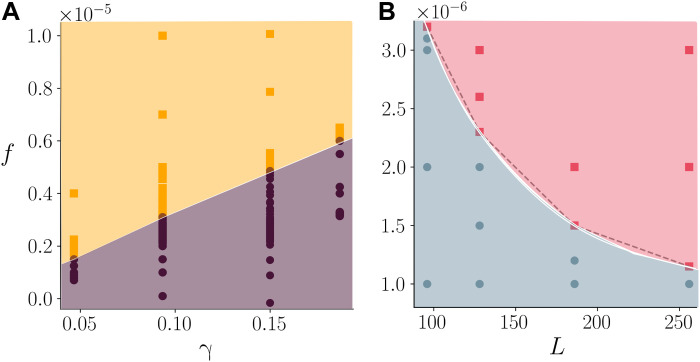
Yielding phase diagram. (**A**) Phase diagram as a function of body-force *f* and surface tension γ. Orange squares, flowing systems; purple circles, nonmoving states. (**B**) Phase diagram as a function of *f* and system size *L* plane. Red squares, flowing systems; blue circles, nonmoving states.

To verify that our qualitative mechanism for yielding through interfacial deformations is correct, we independently varied the parameters in Eq. 1 to see how they affect the value of the critical forcing. We found that *f_c_* scales linearly with surface tension, γ (Fig. 5A), and interfacial width, ξ (see fig. S6). The only other parameters appreciably affecting *f_c_* are the system size *L* and the droplet radius *R*: Increasing either of these lengthscales leads to a decrease in *f_c_* (Fig. 5B). Our data therefore suggest that a key dimensionless parameter may be the capillary number *Ca* = *fLR*^2^/(γξ), which was also empirically found to determine the physics of discontinuous shear thinning (*25*, *26*, *30*). This can be viewed as an inverse Bingham number σ*_v_*/σ*_y_*, with σ*_v_* ∼ *fL* being the viscous stress and σ*_y_* ∼ γξ/*R*^2^ being an effective yield stress. The form of this dimensionless control parameter suggests that, for the suspension to yield, the external forcing has to overcome free energy barriers associated with changes in particle shape, whose cost increases with γ and ξ.

Note that the scaling analysis just performed differs from the one corresponding to the case of foams, valid for larger packing fraction of droplets than considered here, and discussed, for instance, in (*7*, *8*). Those works arrived at a yield stress of σ*_y_* ∼ γ/*R* rather than σ*_y_* ∼ γξ/*R*^2^ as found in our case. The different scaling is due to the fact that, in the foam limit, the only physically relevant length scale is the droplet radius *R*, whereas in our case, the interfacial width plays a crucial role because the droplets jam and stop moving, only because of their mutual overlap. Notwithstanding this key difference, it would be interesting to study the foam limit in more detail numerically and see whether, in that case as well, the yielding transition involves permeation flow and is associated with oscillations and stick-slip motion close to criticality, as in the case we study.

## DISCUSSION

In summary, we studied the rheology of a soft droplet suspension under pressure-driven flow. We found that the droplets only start moving when the forcing that they are subjected to exceeds a critical threshold, as in an ideal yield-stress fluid. However, unlike one such material, even when droplets are jammed, the solvent flows through them via permeation, as in sheared cholesteric (*46*) and smectic liquid crystals (*47*) and fiber gels (*48*), leading to an effective viscosity that depends on the system geometry and ceases to be a bulk property of the material. Yielding is accompanied by a morphological transition. The jammed phase is amorphous, and the network of droplet-droplet contacts, or overlaps, percolates in the direction perpendicular to the wall, conferring rigidity to the system. In the flowing phase, droplets order and contact percolation is lost. Within this picture, overlaps play a qualitatively similar role to frictional contacts in hard colloids (*28*). In our case, however, the transition between the jammed and flowing phase requires a transient change in droplet shape. More quantitatively, yielding occurs for a sufficiently large value of an inverse Bingham number, controlling the balance between viscous and interfacial stresses. The mechanism is therefore similar to that determining discontinuous shear thinning at larger forcing (*25*, *26*, *30*): The fundamental difference is that, at the yielding transition, interfacial deformations are spatially localized and transient in time, whereas at the discontinuous shear-thinning transitions, they affect large portions of the system and occur at all times. Notably, we predict that the yield-stress behavior can be completely eliminated in our model by allowing droplet areas to fluctuate, for instance, because of evaporation/condensation phenomena. We hope that our results will stimulate experiments, e.g., with stabilized oil-in-water or water-in-oil emulsions, to directly test our predictions on the scaling of *f_c_* and on the importance of permeation for the yielding transition. To assess the universality of our results, one could investigate the yielding transition in other materials, such as biological tissues (*33*, *49*, *50*), red blood cell suspensions (*51*), and liquid crystalline emulsions (*52*–*55*). It would also be of interest to revisit the foam limit considered in previous literature (*7*, *8*) to quantify permeation there and to ask whether the nonconserved and conserved model are fundamentally distinct in that limit as well. Last, it would also be desirable to extend our theory to other deformable materials, such as microgels (*31*, *32*, *56*), where Brownian motion is important.

## MATERIALS AND METHODS

### Numerical simulations

To solve the set of partial differential Eqs. 2 and 3 and 3 to 5, we use a hybrid LB algorithm (*38*, *57*). In this framework, the dynamics of the compositional order parameters ϕ*_i_* (with *i* = 1, …, *N*, *N* being the number of droplets) is solved by means of a finite difference algorithm, whereas the Navier-Stokes equation for the incompressible velocity field **v** is solved by a predictor corrector LB. The numerical algorithm has been parallelized by implementing standard domain decomposition with message passing interface (*38*). The pressure gradient is included as a body-force in our LB algorithm, and this is added to the collision operator at each lattice node.

In all the simulations presented here, neutral wetting boundary conditions are enforced. These require that, at the walls

$$\begin{array}{cc} \frac{\partial \mu_{i}}{\partial z} & =0\\ \frac{\partial \nabla^{2}\phi_{i}}{\partial z} & =0 \end{array}\;\text{for each droplet}\;i=1,\ldots ,N$$ where the first line ensures density conservation, while the second determines the wetting to be neutral.

Parameters used in this study are as follows. We fixed the droplet radius to *R* = 8 and the mobility to *M* = 0.1. Unless otherwise specified (like in the case of the phase diagram in Fig. 3), free energy parameters are α = 0.07, *K* = 0.14, and ϵ = 0.05. The nominal viscosity of both the solvent and the fluid inside the droplet is η_0_ = 5/3. Simulations were performed for different system sizes, ranging from *L* = 96 to *L* = 256, maintaining the packing fraction constant to Φ = 0.51. We used periodic boundary conditions along the *y* axis and boundary walls along the *z* axis. Simulations were carried over for at least 10^8^ iterations to guarantee stationarity. This corresponds to runs of 120 hours using 64 cores for systems of size *L* = 128. The parameters listed above can be mapped onto a physical system by fixing the droplet radius *R* = 100 μm, the nominal viscosity η_0_ = 10 cP or 10^−2^ Pa·s, and surface tension $\gamma =\sqrt{\left(8K\alpha /9\right)}∼0.09$ to 1 mN/m. With this mapping, a velocity of 10^−3^ in simulation units (lattice units) corresponds to 1 mm/s. The Reynolds number range from ∼0.4 to ∼8. The conventional capillary number, defined as $\mathrm{Ca}=\frac{\eta_{0}v}{\gamma }$ (with η_0_ being the solvent viscosity and *v* = *v_max_*), ranges between ∼0.04 (for *f* = 1.0 × 10^−6^) and ∼1.23 (for *f* = 10^−4^). The density-based spatial clustering analysis used to produce the insets of Fig. 1 has been carried out using the DBSCAN algorithm (*58*) on the free energy of overlaps.
