# Supplementary material for: Yield-stress transition in suspensions of deformable droplets
Source: Sci Adv. 2023 May 31;9(22):eadf8106. doi: 10.1126/sciadv.adf8106 (PMC10413676; doi:10.1126/sciadv.adf8106)
Supplement: Supplementary file 1 — Figs. S1 to S7 Legends for movies S1 to S4 [file sciadv.adf8106_sm.pdf]

Science Advances
AAAS

# Supplementary Materials for

## Yield-stress transition in suspensions of deformable droplets

Giuseppe Negro *et al.*

Corresponding author: Giuseppe Negro, giuseppe.settimio.negro@gmail.com

*Sci. Adv.* **9**, eadf8106 (2023)
DOI: 10.1126/sciadv.adf8106

**The PDF file includes:**

Figs. S1 to S7
Legends for movies S1 to S4

**Other Supplementary Material for this manuscript includes the following:**

Movies S1 to S4

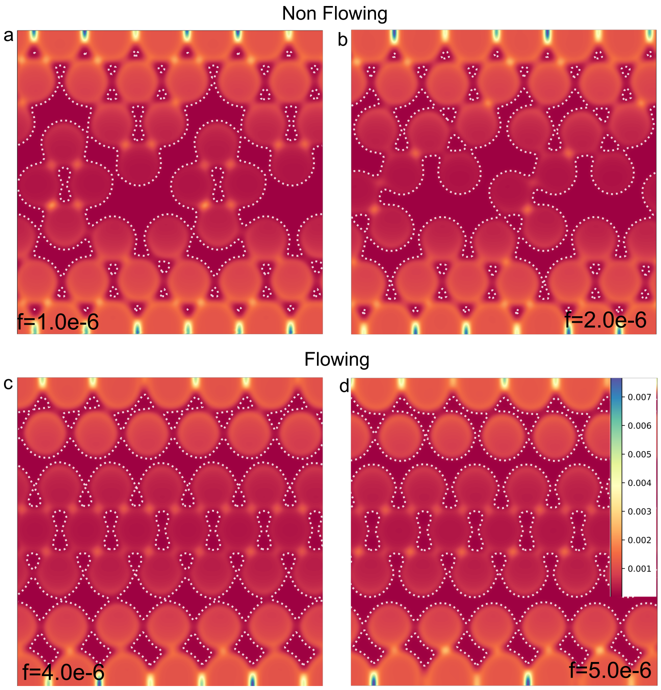


FIG. S1. **Overlap free energy before and after the yielding transition.** Color maps of the free energy of overlaps $f_{\text{overlaps}}$ defined in the main text, for different values of body force $f$ before ((a)-(b)) and after ((c)-(d)) the yielding transition . White contour lines correspond to $f_{\text{overlaps}} = 0.001$. For low values of the bodyforce $f$ ((a)-(b)) droplet contacts percolate between the two walls. Beyond the transition the pattern of droplet contacts no longer percolate in the flow gradient direction.

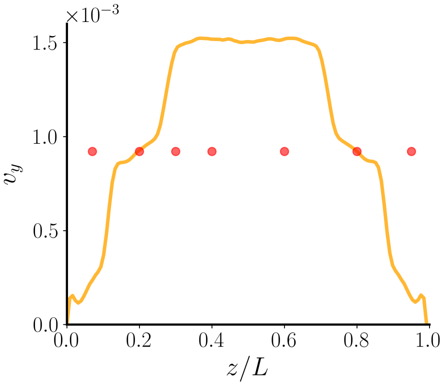


FIG. S2. **Plug-like flow behaviour.** Fluid velocity profile (as a function of the channel width $z/L$), for $f = 4.0 \times 10^{-10}$, beyond the yielding transition. The flow appears to be plug-like, with the velocity of the droplets (solid circles) being less than the overall fluid velocity, showing that the flow has a permeative component.

## ADDITIONAL RESULTS

In the following paragraphs we provide some additional evidence to support the results presented in the main text.

### Morphology and flow behaviour near the yield-stress transition

As mentioned in the main text (see Fig. 1 and related discussion) the yielding transition is accompanied by a morphological transition, from a non-flowing, disordered state to a flowing, ordered one. In the former, droplets overlaps create a percolating network whereas after yielding the pattern of droplet contacts no longer percolate along the flow gradient direction. This change can be appreciated by looking at the color maps of the free energy of overlaps (computed in steady state), for different values of the body-force $f$, which are displayed in Fig. S1. Moreover, in the flowing state the flow becomes plug-like (see Fig. S2).

To better understand the morphological differences between the droplets configurations before and after the yielding transition we computed the distribution of the area of the Voronoi cells calculated by starting from the droplet centres of mass. The results, for two configurations before ($f = 0.5 \times 10^{-6}$) and after the transition ($f = 5.5 \times 10^{-6}$), are displayed in Fig. S3(a) and (b), respectively. Although no defects are present in the Voronoi tessellation in either cases , the Voronoi cells sensibly deform in the non-flowing configuration (panel (a)) while they arrange in a more regular shape in the flowing state (panel (b)). This behaviour is quantified by the distribution of the area of the Voronoi cells in the two cases (panel(c)).

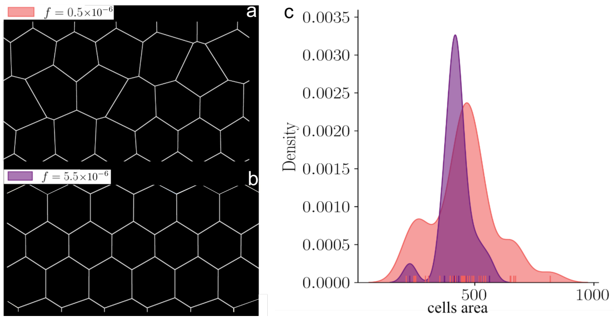


FIG. S3. **Voronoi tessellation and corresponding area distribution. (a)-(b)** Results of a Voronoi tessellation, performed on the bulk of the system, for two values of the body-force in the non-flowing state ((a) for $f = 2.0 \times 10^{-6}$) and in the flowing state ((b) for $f = 4.5 \times 10^{-6}$). Panels (a) and (b) correspond to the configurations shown in Fig.1 in the main text. **(c)** Distribution of the area of the Voronoi cells for the two configurations shown in panel (a) and (b). In the non-flowing state the distribution of the area is spread over a wider range of values and shows secondary peaks corresponding to a more irregular droplet arrangement.

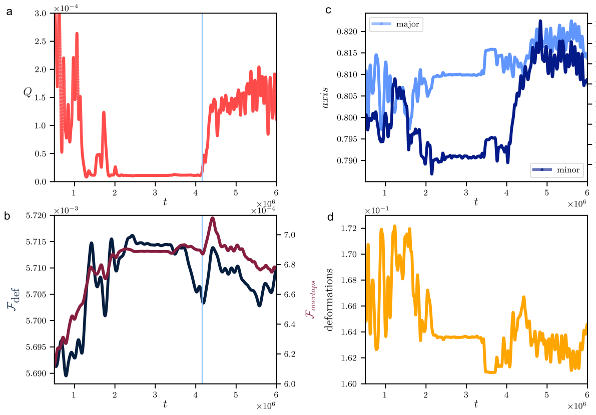


FIG. S4. **Stick-slip motion.** The figures shows the time series of some relevant observables computed for the $f = 3.5 \times 10^{-6}$, close to the yielding transition, where we observe the intermittent stick-slip motion discussed in the text. **(a)** Throughput flow $Q$. **(b)** Elastic energy ($\sim K$) measuring droplet deformation (dark blue curve) and overlap free energy ($\sim \epsilon$) measuring droplet response to contacts (purple curve). **(c)** Major (dark blue) and minor (light blue) axis ($d_{max}$ and $d_{min}$, respectively) computed as eigenvalues of the intertial tensor (see text) normalized with respect to the nominal droplet radius $R$. **(d)** Droplet deformation computed as the relative difference of the major and minor axis $(d_{max} - d_{min})/(d_{max} + d_{min})$. The vertical line at iteration $t = 4.13 \times 10^6$ marks the time at which a slipping events occurs.

## Stick-slip motion

Close to the transition point $f_c$ the dynamics of the system is characterized by the stick-slip motion of the droplets, namely an intermittent regime where the

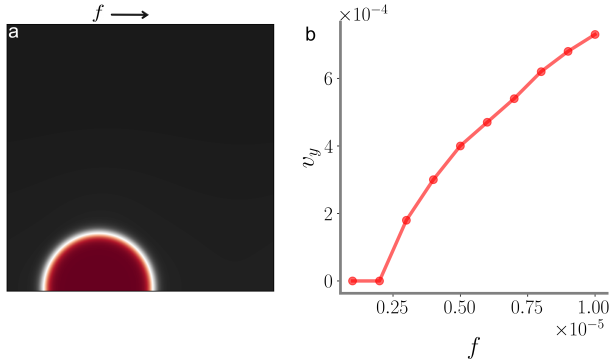


FIG. S5. **Single droplet. (a)** System set-up for the case of a single droplet at a solid wall under an external forcing, with neutral wetting boundary conditions at the wall. Note there is also a no-slip wall on the top boundary. **(b)** $y$ component of the droplet velocity as a function of the body-force $f$ for a single droplet in the conserved model, exhibiting yield-stress behavior.

droplets alternate stationary lapses –with the droplets sticking close to each others– and bursts of motion where the accumulated stress relaxes, leading the system of droplets to move and arrange in a new stationary configuration. These observations can be rationalized by looking at the time series of some dynamical observables shown in Fig. S4. Panel (a) shows the total throughput $Q$ as a function of time. After the initial settling dynamics, the throughput drops orders of magnitude, signalling that the system only features permeative flows, typical of the solid-like phase. At time $t = 4.13 \times 10^6$, the layers of droplets in the bulk *slip* along those anchored to the boundary (see movie S4), resulting in a steep increase of the throughput flow. It is interesting to compare this behavior with the free energy evolution in panel (b). Along the preceding stationary lapse, both the elastic free energy $\mathcal{F}_{def}$ (dark blue line) and the overlap free energy $\mathcal{F}_{overlap}$ (purple line) are first constant and begin to sensibly decline as the slipping event is approached. This occurs as the result of small rearrangements of the droplets in the channel responsible for the release of the excess stress. As the droplets begin to move, both terms rapidly increase again, both because the moving droplets in the bulk tend to push on each other causing overlaps, and because the motion of the droplets is also accompanied by their deformation.

To quantify the droplet deformation we measure the major and minor axis of the droplets, $d_{max}$ and $d_{min}$, respectively. These can be calculated, by computing the square root of the two eigenvalues of the tensor of inertia:

$$I_i = \frac{\int d\boldsymbol{r}'\phi_i(\boldsymbol{r}')\boldsymbol{r}'\boldsymbol{r}'}{\int d\boldsymbol{r}'\phi_i(\boldsymbol{r}')} \ , \qquad \text{for every droplet } i = 1, \ldots, N,$$

$\boldsymbol{r}'$ expressed in the reference system of the center of mass of the $i$-th droplet. Finally, the deformation of the

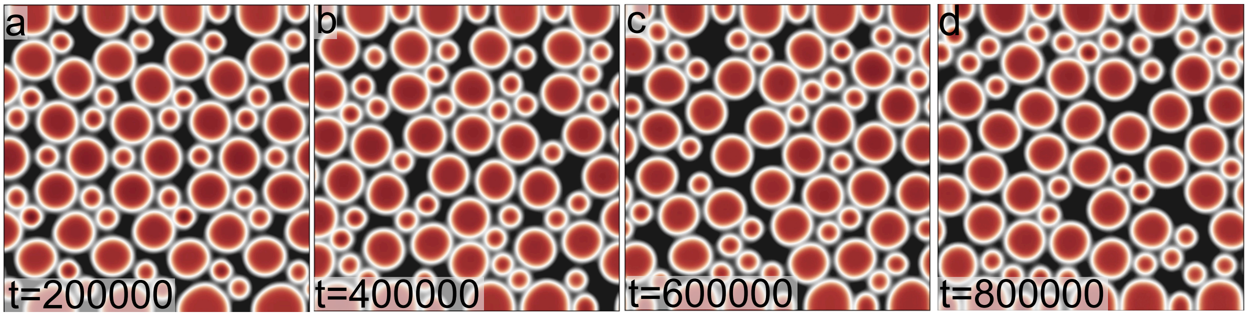


FIG. S6. **Polydisperse suspensions. (a)-(d)** Snapshots of the system during time evolution, after the yielding transition ($f = 6.0 \times 10^{-6}$), showing the segregation of small and big droplets discussed in the main text.

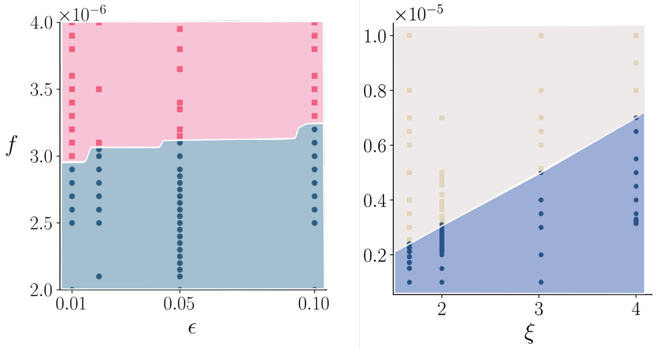

FIG. S7. **Phase diagram of the yield-stress transition for the conserved model. (left)** Phase diagram in the body-force $f$ versus droplets repulsion parameter $\epsilon$ plane. Red squares denote a flowing system, while blue circles non-flowing ones. **(right)** Phase diagram in the body-force $f$ versus interface thickness $\xi$ plane. Ivory squares denote a flowing system, while blue circles non-flowing ones. While the yielding transition is only slightly affected by $\epsilon$, the threshold force $f_c$ scales linearly with the $\xi$.

droplet can be measured as the relative difference of the major and minor axis $(d_{max} - d_{min})/(d_{max} + d_{min})$. The behavior of both the droplet axis and deformation, for a droplet in the bulk, is shown in panels (c) and (d), respectively. It is worth noting that right before the slipping event the droplet deformation is first drastically reduced, then it increases again as the system starts to flow.

### Single Droplet

As mentioned in the main text, more insights into the fundamental difference in behaviour between the conserved and non-conserved model can be gained by analysing in more detail the behaviour of a single droplet at a solid wall under an external forcing, and with neutral wetting boundary conditions (Fig. S5(a)). In the conserved model the droplet sticks to the wall and requires a finite forcing to start moving, as showed by the plot of the $y$-component of the droplet velocity as a function of the body-force $f$, in Fig. S5(b). In the non-conserved model, instead, evaporation and condensation provide also in this case another pathway for contact line motion, and the droplet drifts along the wall for any value of the forcing. All parameters are the same as in the multiphase simulations except for the droplet radius which is $R = 8.5$. and the simulation box which is of size $L = 96$.

### Polydisperse suspensions

In the main text we explored the effect of polydispersity on the yielding transition by considering a bi-disperse suspension (where the droplet radius of one component is twice as large as that of the other). Analogously to what observed in the monodisperse case, for low values of the bodyforce, the suspended droplets are jammed and immobile (panels (a) and (c) of Fig. 4 of the main text). As the bodyforce is increased, a transition to a flowing state is observed (panels (b) and (c) of Fig. 4 of the main text). The transition is accompanied by a morphological rearrangement with the droplets of the two species separating in different region of the channel, with the large droplets in the centre of the channel, and the small ones close to the walls, effectively creating a lubricating layer. The time evolution of the system, after the yielding transition, is shown in Fig. S6.

### Parameter dependencies

To dissect the fundamental mechanisms leading to the existence of a yield stress in our suspensions and determining its value, we independently varied the parameters in our system to see how this affects the location of the critical force. In Fig. 3 of the main text, the phase diagram is shown in two planes – body-force $f$ versus surface tension $\gamma$, and body-force system versus size $L$. To complete the parameter scanning we show in Fig. S7 the phase diagrams of the yield-stress transition obtained changing the droplets repulsion parameter $\epsilon$ and the interface thickness $\xi$. While the yielding transition is only

slightly affected by $\epsilon$, the threshold force $f_c$ scales linearly with the $\xi$. The latter result support the claim made in the main text, that a key dimensionless parameter in our system is the capillary number $Ca = fLR^2/(\gamma\xi)$.

### Movies Description

Here we describe the movies cited in the main paper. All the movies shown are obtained from numerical simulations of the conserved model.

- **Movie S1**. **Before the Yielding Transition: Jammed Non-flowing State.** The Movie shows the time evolution of $\phi = \sum_i \phi_i$, where black and red regions correspond to $\phi = 0$ and $\phi = 2$ respectively, for $f = 2.0 \times 10^{-6}$. During the system evolution droplets remains essentially immobile, except for small rearrangements resulting in the creation of a network of contacts which percolates along the direction perpendicular to the imposed flow (Fig.1 of the main text). This behaviour is archetypal of the response of the system under study under very small values of imposed forcing.
- **Movie S2. After the Yielding Transition: Unjammed Flowing State.** The Movie shows the time evolution of $\phi$ for $f = 3.8 \times 10^{-6}$. In this case droplets flow under external forcing.
- **Movie S3. Single Droplet attached to the wall: Stick-Slip Motion**. The Movie shows the time evolution of $\phi$, for the case of a single droplet attached to a wall with neutral wetting boundary conditions, for $f = 2 \times 10^{-6}$. The droplet undergoes a stick-slip motion.
- **Movie S4. Stick-Slip Motion near the Yielding Transition.** The movie shows the time evolution of $\phi$ for $f = 3.5 \times 10^{-6}$, near the yielding transition, corresponding to the case considered in Fig. S4. The initial part of the time evolution is characterized by an intermittent regime where the droplets alternate stationary lapses – with the droplets sticking close to each others — and bursts of motion where they steadily move. This is the behaviour referred to as stick-slip motion in the text. After this first regime droplets remain immobile for a very long time before a rearrangement of some droplets (see those highlighted with a violet box) causes the system to flow again.
